# Autistic and schizotypal traits exhibit similarities in their impact on mentalization and adult attachment impairments: a cross-sectional study

**DOI:** 10.1186/s12888-024-06048-9

**Published:** 2024-10-03

**Authors:** Dániel Sörnyei, Ágota Vass, Dezső Németh, Kinga Farkas

**Affiliations:** 1 Department of Psychiatry and Psychotherapy, Semmelweis University, Balassa utca 6, Budapest, 1083 Hungary; 2 Department of Clinical Psychology, Semmelweis University, Üllői út 25, Budapest, 1091 Hungary; 3 Institute of Psychology, ELTE Eötvös Loránd University, Izabella utca 46, Budapest, 1064 Hungary; 4 Centre de Recherche en Neurosciences de Lyon CRNL U1028 UMR5292, INSERM, Université Claude Bernard Lyon 1, CNRS, Bron, France; 5 NAP Research Group, Institute of Psychology, Eötvös Loránd University & Institute of Cognitive Neuroscience and Psychology, HUN-REN Research Centre for Natural Sciences, Budapest, Hungary; 6 Department of Education and Psychology, Faculty of Social Sciences, University of Atlántico Medio, Las Palmas de Gran Canaria, Spain

**Keywords:** Autism, Mentalizing, Schizophrenia, Schizotypy, Social support, Transdiagnostic

## Abstract

**Background:**

Deficits in mentalizing and attachment occur in the autism and schizophrenia spectrum, and their extended traits in the general population. Parental attachment and the broader social environment highly influence the development of mentalizing. Given the similarities in the symptomatology and neurodevelopmental correlates of autism spectrum disorder (ASD) and schizophrenia (SCH), it is crucial to identify their overlaps and differences to support screening, differential diagnosis, and intervention.

**Methods:**

This cross-sectional study utilized questionnaire data from 2203 adults (65.1% female, mean age[SD] = 37.98[9.66]), including participants diagnosed with ASD, SCH, and those exhibiting subclinical traits to investigate the associations between mentalizing, attachment, and perceived social support during adolescence across the autistic and schizotypy spectrum.

**Results:**

It was revealed that both autistic and schizotypal traits have comparable effects on insecure adult attachment, primarily through challenges in mentalizing. The impact of mentalizing deficits on adult attachment slightly varies between autistic and schizotypal traits. Conversely, perceived social support during adolescence relates to improved mentalizing and secure adult attachment as a protective factor during development.

**Conclusions:**

These outcomes highlight the significance of supportive therapeutic relationships and community care while suggesting directions for further research and collaborative treatments addressing ASD and SCH, considering the differential impact of mentalizing on adult attachment.

## Background

Autism spectrum disorder (ASD) is a neurodevelopmental condition characterized by difficulties in reciprocal social interaction, communication, and restricted, repetitive patterns of behavior that can be detected early in life and persist to adulthood [1]. Schizophrenia (SCH) is a complex neuropsychiatric disorder defined by *positive* (delusions and hallucinations), *negative* (social and emotional withdrawal, apathy), and *disorganized* (thought, speech, and motor behavior disruption) symptoms [1]. Although the first psychotic episode in SCH typically occurs in early adulthood, subtle alterations in cognitive and motor functions are present much earlier, indicative of altered neurodevelopment [2, 3]. Both ASD and SCH can be conceptualized as extended endophenotypic dimensions, referred to as autistic [4, 5] and schizotypal [6, 7] traits. It means that the features of ASD and SCH are widely present in the general population, ranging from very mild forms to subclinical presentations and extreme cases requiring therapeutic intervention, reflecting a liability to disorder.

Recently, numerous studies have pointed out significant overlaps in the prevalence and symptomatology (e.g., social and communication discomfort, relationship disinterest, and difficulty interpreting social cues) of ASD and SCH, hindering differential diagnosis, especially in adulthood [8–13]. Notably, the prevalence of SCH and other psychotic disorders among individuals with ASD has been reported to range from 4 to 67% [14–16]. The reported prevalence rates of diagnosed ASD in individuals with psychotic disorders range from 0.78 to 52%, while the prevalence of autistic-like traits in this population ranges from 9.6 to 61% [11]. Moreover, the two disorders share genetic and neurodevelopmental correlates as well as environmental risk factors, and their co-occurrence is associated with worse clinical outcomes [17–21]. Some theories suggest a common vulnerability model for ASD and SCH, with *aberrant salience*,* asociality* and *concrete thinking* being the common underlying dimensions [22], while others conceptualize ASD and SCH as diametric disorders of the social brain [23, 24]. As autistic and schizotypal traits overlap significantly, and a great portion of clinically affected individuals are only diagnosed in adulthood, it is crucial to examine the two sets of features in the same sample of adults to support differential diagnosis, develop novel screening tools, and identify possible targets of early intervention. Here, our objective is to explore mentalizing and attachment, which could potentially unlock crucial insights into the partially shared developmental origins of ASD and SCH, as their underlying neural deviations affect the mentalizing network in the brain [19]. Our dimensional approach aligns with the broader movement in psychopathology research, as continuous measures have been shown to exceed the reliability and validity of discrete diagnostic categories [25, 26].

Mentalizing is the human-specific, social-cognitive ability to attribute different mental states (e.g., thoughts, goals, desires, intentions, emotions) to oneself and others [27], which is essential to navigating everyday social interactions. It is a multifaceted concept that encompasses four dimensions [28]: (1) mentalizing can be automatic or controlled; (2) it can refer to our own mental states or those of others; (3) it can be external or internal; and (4) cognitive or affective. The neural bases of mentalizing involve complex cortical and subcortical networks. Prefrontal cortical areas play a crucial role in cognitive-controlled mentalizing, whereas parietal cortical and subcortical circuits are involved in automatic-affective processes, with the amygdala being of central importance [29]. It is widely accepted that mentalizing deficits underlie social difficulties (e.g. in social-emotional reciprocity, non-verbal communication, and maintaining relationships) observed in autism [30–32]. Difficulties in mentalizing are also well-documented in SCH [33, 34], such as paranoid delusions which can be considered distortions of reality resulting from impaired mentalizing of the self [35]. Recent systematic reviews and meta-analyses have found that difficulties in cognitive mentalizing, emotional intelligence, and social skills in ASD are comparable to those observed in SCH [36–38]. However, differences emerge across age groups, with social and non-social cognitive abilities being more impaired in SCH than in ASD, particularly in the later years of young adulthood [39]. Furthermore, recent dimensional studies have found that autistic and schizotypal traits are similarly linked to poorer social functioning, particularly in the case of autistic traits and negative schizotypy [40, 41]. Moreover, previous studies have found that mentalization-based interventions can improve social functioning in individuals with psychotic disorders [42, 43], and there is preliminary evidence for the efficacy of these interventions in autism as well [44, 45].

The development of mentalizing is highly impacted by social and environmental factors, particularly relationships with other individuals [46], such as secure attachment with the primary caregiver, since internal working models [47] formed within this relationship provide the representational basis for understanding others. In this context, *epistemic trust* is a key concept, which refers to our innate ability to perceive others as reliable sources of social information. This facilitates the development of flexible mentalizing and stable adult attachments [28]. In adulthood, close relationships with others are described along the dimensions of *proximity*,* dependence*, and *anxiety*, and categorized into *secure*,* avoidant*, and *anxious/ambivalent* attachment styles [48, 49]. Securely attached adults maintain comfortably balanced closeness in intimate relationships, without being anxious about proximity or distance. Avoidant individuals, on the other hand, experience anxiety in intimacy, therefore they typically tend to avoid close relationships. Those with an anxious/ambivalent attachment style have a high need for proximity, while also frequently experiencing fear of abandonment and rejection. The current developmental psychopathology framework of mentalizing considers not only the early parent-child dyadic relationship but also the influence of family, peers, and the broader sociocultural environment [28]. To our knowledge, no study has specifically examined the relationship between mentalizing, adult attachment, and perceived social support. However, higher levels of perceived social support have been linked to increased positive affect, life satisfaction, and favorable outcomes of mental disorders [50–52]. Thus, we hypothesized that the amount of perceived social support received from one’s environment is an additional key factor in social-cognitive development, specifically playing a protective role against mentalizing difficulties and insecure attachment.

Although deficits in mentalizing are well-documented and consistently observed in individuals with both ASD and SCH, there have been only a few studies investigating attachment styles. In particular, there is a paucity of research examining the autism and schizophrenia spectrum together in adult populations. Two earlier meta-analyses found that children with ASD exhibit a higher proportion of insecure and disorganized attachment styles compared to their neurotypical peers [53, 54]. Another study [55] reported similar findings in a cross-sectional study of attachment styles among adults with ASD. In a non-clinical sample of adults, it was found that the relationship between autistic traits and attachment avoidance is mediated by lower empathy [56]. Similarly, autistic traits were specifically linked to attachment avoidance in a cross-sectional study of university students [57]. Finally, a recent study indicated that higher attachment anxiety and avoidance mediate the relationship between autistic traits and lower relationship satisfaction [58]. Previous meta-analyses have also highlighted a significant association between psychotic symptoms and insecure attachment styles [59]. Furthermore, psychotic experiences were linked to insecure attachment in clinical, high-risk, and non-clinical samples as well [60, 61]. Overall, it appears that difficulties in attachment and mentalizing might be considered transdiagnostic factors of autism and schizophrenia spectrum.

**Fig. 1:**
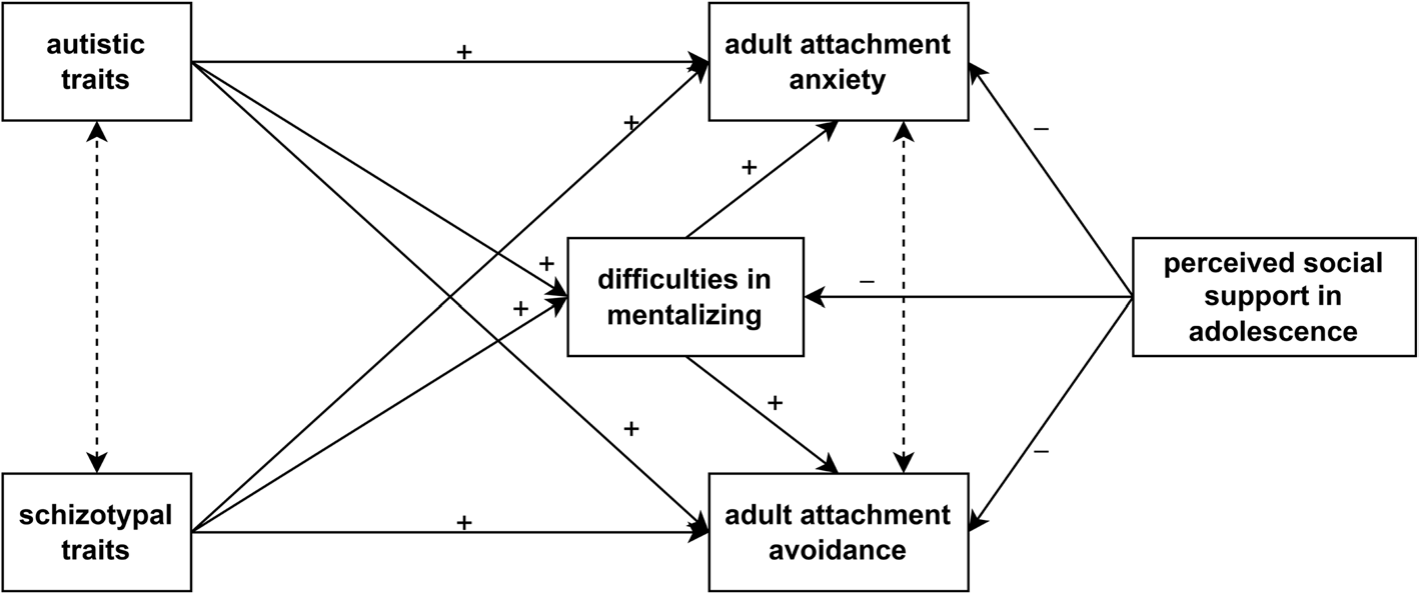
Proposed relationships between constructs. Note: Dashed, bidirectional arrows indicate correlational effects, while continuous, unidirectional arrows denote direct and indirect regression effects. We hypothesized that autistic traits contribute to higher levels of adult attachment anxiety and avoidance, mediated by difficulties in mentalizing. Similarly, we proposed that schizotypal traits (specifically, positive, negative, and disorganized schizotypy) predict adult attachment anxiety and avoidance via difficulties in mentalizing. Furthermore, we expected that perceived social support in adolescence is negatively associated with difficulties in mentalizing as well as adult attachment anxiety and avoidance

In this study, our main goal was to investigate the characteristics of attachment and mentalizing in relation to autistic and schizotypal traits, which may be key to understanding the partially common developmental roots of ASD and SCH as manifest psychiatric conditions. The relationship is likely bidirectional and rather complex: autistic and schizotypal traits might both influence and be influenced by attachment styles and mentalizing abilities, suggesting a dynamic interplay rather than a straightforward causal pathway. While secure attachments (both in childhood and adulthood) provide a foundational model for understanding others, mentalizing abilities can also influence the formation and quality of these attachments. However, given the cross-sectional nature of the study, causality cannot be established, and the research focuses on adult attachment styles as outcomes in this interaction. Figure 1 represents the proposed relationships between constructs based on the literature. Combining the research findings presented above, we proposed the following hypotheses: (1) Autistic traits contribute to higher levels of adult attachment anxiety and avoidance, mediated by difficulties in mentalizing [55–58]. Similarly, we presumed that (2) schizotypal traits are associated with higher levels of adult attachment anxiety and avoidance, mediated by difficulties in mentalizing [35, 59–61]. Furthermore, we hypothesized that (3) perceived social support in adolescence is negatively related to difficulties in mentalizing, as well as adult attachment anxiety, and avoidance [28, 50–52].

## Methods

### Procedure

The language of the study was Hungarian. For data collection, we used online self-report questionnaires, in a cross-sectional design. Our participants were recruited through various channels, including social media platforms (e.g., Facebook advertisements), outpatient units, and organizations working with ASD individuals (via flyers and caregiver recommendations). Potential participants could contact the research team via email or through a direct link and QR code that led to the questionnaire battery, which was created using formr.org [62], an online survey framework. The advertisements and flyers also included an email address (specifically created for this research project) that participants could use to ask questions and seek further (medical) assistance if needed. Participants completed the survey through the formr.org [62] website, as part of a larger battery of tests. The process began with providing voluntary, informed consent to participate, followed by the questionnaires. The procedure complied with the ethical standards of national and institutional committees on human experimentation and with the Helsinki Declaration.

### Participants

A total of 2762 individuals agreed to complete the survey, out of which 2269 completed the Mentalization Questionnaire following the items relating to demographic data. Participants were recruited through social media platforms, outpatient units, and organizations working with ASD individuals, using convenience sampling. To ensure the high quality of our data and the authenticity of participants, we have constructed three criteria. First, we excluded participants who did not complete the Mentalization Questionnaire, as it was essential for measuring difficulties in mentalizing, a core variable of our mediation models. Second, we included only individuals aged between 18 and 65 years (*N* = 2225). Finally, we confirmed that all participants had completed at least eight years of elementary education or provided realistic data regarding their educational history, excluding those who reported more than 30 years of education (*N* = 2203). In order to explore the entire continuum of autistic and schizotypal traits, no participants were excluded based on their psychiatric diagnosis. Thus, the final sample consisted of 2203 participants, of whom 711 (32.3%) were male, 1434 (65.1%) were female, and 58 (2.6%) identified as another gender. Detailed demographics are presented in Table 1. Informed, voluntary consent was obtained from all participants. The study was conducted in accordance with the Helsinki Declaration, and it was approved by the Semmelweis University Regional and Institutional Committee of Science and Research Ethics (approval number: RKEB 158/2021).

**Table 1:** Detailed demographics of the final sample (*N* = 2203)

Gender (*N* [%])	Male	Female	Other							
	711 [32.27%]	1434 [65.09%]	58 [2.63%]							
Diagnosis (*N* [%])	ASD	SCH	AQ-50 score ≥ 32*	AQ-50 score < 32*	AQ-50 score≥ 26**	AQ-50 score < 26**	Missing AQ-50 score			
	172 [7.81%]	25 [1.13%]	320 [14.52%]	1550 [70.36%]	639 [29.00%]	1231 [55.88%]	333 [15.12%]			
	Minimum	Maximum	Mean	Median	Standard deviation	Skewness				
Age (years)	19	65	37.98	38	9.66	0.25				
Education (years)	8	30	17.67	18	3.44	0.41				
Work status (*N* [%])	Student	Full-time (white c.)	Part-time (white c.)	Full-time (blue c.)	Part-time (blue c.)	Parenting	Home care	Pensioner	Unemployed	Unknown
	193 [8.76%]	1328 [60.28%]	202 [9.17%]	130 [5.90%]	31 [1.41%]	153 [6.95%]	37 [1.68%]	32 [1.45%]	95 [4.31%]	2 [0.09%]
Residence (*N* [%])	Metropolitan> 1 M	Large city100,000 – 1M	Medium city20,000 – 100,000	Small city 5000 – 10,000	Village1000 – 5000	Small village < 1000	Farm			
	1219 [55.33%]	278 [12.62%]	280 [12.71%]	216 [9.81%]	159 [7.22%]	50 [2.27%]	1 [0.05%]			
Category of education (*N* [%])	PhD/postgrad	MA/MSc	BA/BSc	National Qualification Register	Graduation	Vocational	Elementary	Unknown		
	165 [7.49%]	792 [35.95%]	614 [27.87%]	247 [11.21%]	319 [14.48%]	43 [1.95%]	22 [1.00%]	1 [0.05%]		
Socioeconomics (*N* [%])	Own property	Family property	Rental	Student hall	Social institute	Homeless	Other	Not disclosed		
	1046 [47.48%]	586 [26.60%]	507 [23.01%]	21 [0.95%]	1 [0.05%]	0 [0.00%]	30 [1.36%]	12 [0.55%]		
Marital status (*N* [%])	Single	Relationship	Married	Divorced	Widowed	Other				
	633 [28.73%]	579 [26.28%]	833 [37.81%]	116 [5.27%]	15 [0.68%]	27 [1.23%]				

*ASD* psychiatric diagnosis of autism spectrum disorder, *SCH* psychiatric diagnosis of schizophrenia spectrum disorder, *AQ-50* Autism-Spectrum Quotient. *AQ-50 scores of 32 and higher indicate high levels of autistic traits as per Baron-Cohen et al. [63]; **AQ-50 scores of 26 and higher indicate high levels of autistic traits as per Woodbury-Smith et al. [64]

### Measures

#### Autism spectrum quotient (AQ-50)

The Autism-Spectrum Quotient (AQ-50; [63]) was utilized for the assessment of autistic traits. This self-report questionnaire was developed for screening purposes for adults in the average intelligence range. The AQ-50 measures the occurrence of autistic traits across five different domains (*social skill*,* attention switching*,* attention to detail*,* communication*, and *imagination*), each consisting of 10 items. Although a Hungarian adaptation has not yet been developed, the reliability and validity of AQ-50 have been demonstrated in numerous studies abroad [4, 65, 66]. In the original version, Baron-Cohen and colleagues [63] reported Cronbach’s α values ranging from 0.63 to 0.77 for the subscales. In the present study, the reliability indices of the AQ-50 demonstrated satisfactory results (Cronbach’s α = 0.887; McDonald’s ω = 0.889).

#### Multidimensional schizotypy scale – brief (MSS-B)

To examine schizotypal traits, we used the Multidimensional Schizotypy Scale – Brief (MSS-B; [67]). The questionnaire has three subscales corresponding to the main symptom domains of SCH (*positive*,* negative*, and *disorganized*). The total score of the questionnaire cannot be reliably applied, thus the use of separate scores for each of the three subscales is warranted (Cronbach’s α = 0.8–0.9). During the Hungarian adaptation, satisfactory reliability indices were found in a sample of healthy adults (Cronbach’s α = 0.76–0.87) [68]. In the present study, we obtained comparable reliability for the *positive* (Cronbach’s α = 0.781; McDonald’s ω = 0.779), *negative* (Cronbach’s α = 0.817; McDonald’s ω = 0.823), and *disorganized* (Cronbach’s α = 0.891; McDonald’s ω = 0.891) schizotypy scales.

#### Mentalization questionnaire (MZQ)

The Mentalization Questionnaire (MZQ; [69]) measures various difficulties in mentalizing along four subscales (*refusing self-reflection*,* emotional awareness*,* psychic equivalence mode*, and *regulation of affect*). In the original study, the MZQ demonstrated high reliability (Cronbach’s α = 0.81). The questionnaire was adapted to Hungarian on a sample of individuals affected by psychotic disorders, confirming the original four-factor structure (Cronbach’s α = 0.7–0.9) [70]. In the present study, we obtained satisfactory reliability scores (Cronbach’s α = 0.859; McDonald’s ω = 0.865).

#### Adult attachment scale (AAS)

The Adult Attachment Scale (AAS; [48]) measures differences in attachment security in intimate relationships during adulthood. The original questionnaire can be divided into subscales of *close*,* depend*, and *anxiety.* However, in the Hungarian version, the alternative factors of *attachment avoidance* and *attachment anxiety* proved reliable (Cronbach’s α = 0.77–0.87) [71]. In the present study, the subscales of *attachment avoidance* (Cronbach’s α = 0.857; McDonald’s ω = 0.871) and *attachment anxiety* (Cronbach’s α = 0.883; McDonald’s ω = 0.886) demonstrated satisfactory reliability.

#### Multidimensional scale of perceived social support (MSPSS)

The measurement of perceived social support during adolescence was conducted using a slightly modified version of the Multidimensional Scale of Perceived Social Support (MSPSS; [72]). In the original instructions, participants were asked to assess the perceived adequacy of social support in a general context. However, in our questionnaire, we assessed the level of perceived social support received during adolescence, specifically between the ages of 14 and 18, aiming to evaluate the extent to which *family members*,* friends*, and *significant others* were perceived as providing psycho-social resources to the individual. The reliability indices of the questionnaire were found to be satisfactory both in the original study (Cronbach’s α = 0.88) and in the Hungarian adaptation (Cronbach’s α = 0.91) [73]. In the present study, the reliability of the MSPSS also demonstrated excellent values (Cronbach’s α = 0.946; McDonald’s ω = 0.946).

### Statistical analyses

Statistical analyses were conducted using JASP 0.16.1 [74]. Data visualization was performed with R [75], RStudio [76] and the packages *corrplot* [77], *ggdist* [78], *ggthemes* [79], *Hmisc* [80], *patchwork* [81], and *psych* [82]. The variables consisted of aggregated scores of the questionnaires (for distributions see Fig. 4). Based on Shapiro-Wilk tests (all *p* < 0.001) and Q-Q plots, the distribution of the variables did not appear to be normal. Consequently, Spearman’s rank correlation was employed to create a correlation matrix (Fig. 5) between variables, and false discovery rate (FDR) correction was applied to mitigate Type I errors.

**Fig. 2:**
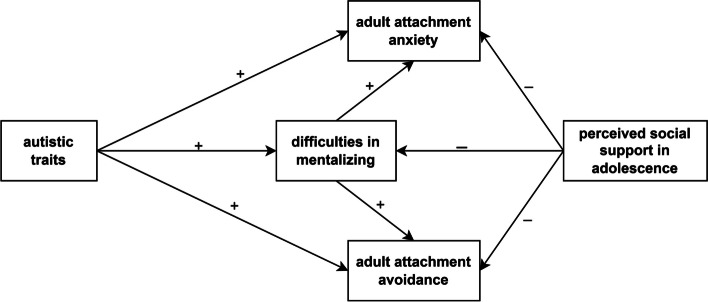
Model A to test the mediating effects of difficulties in mentalizing. Note: Arrows indicate hypothesized direct and indirect effects. Input variables include autistic traits and perceived social support in adolescence. Outcome variables include adult attachment anxiety and avoidance. Control variables such as gender, age, and years of education are not shown in the figure to maintain readability. We hypothesized that autistic traits contribute to higher levels of adult attachment anxiety and avoidance via difficulties in mentalizing. Conversely, perceived social support in adolescence was assumed to be negatively associated with difficulties in mentalizing as well as attachment anxiety and avoidance

We attempted to test our hypotheses using two multivariate mediation models. The separate testing of the two models was justified due to the high multicollinearity between AQ-50 scores and the negative and disorganized subscales of MSS-B, which likely would have resulted in a negative suppressor effect between the predictor variables. In Model A (Fig. 2), we tested the mediating effect of difficulties in mentalizing (MZQ total score), with autistic traits (AQ-50 total score) and perceived social support in adolescence (MSPSS total score) included as predictors, and adult attachment anxiety and avoidance (AAS scores) as outcome variables. To control for potential confounding effects, gender, age, and years of education were included as covariates.

**Fig. 3:**
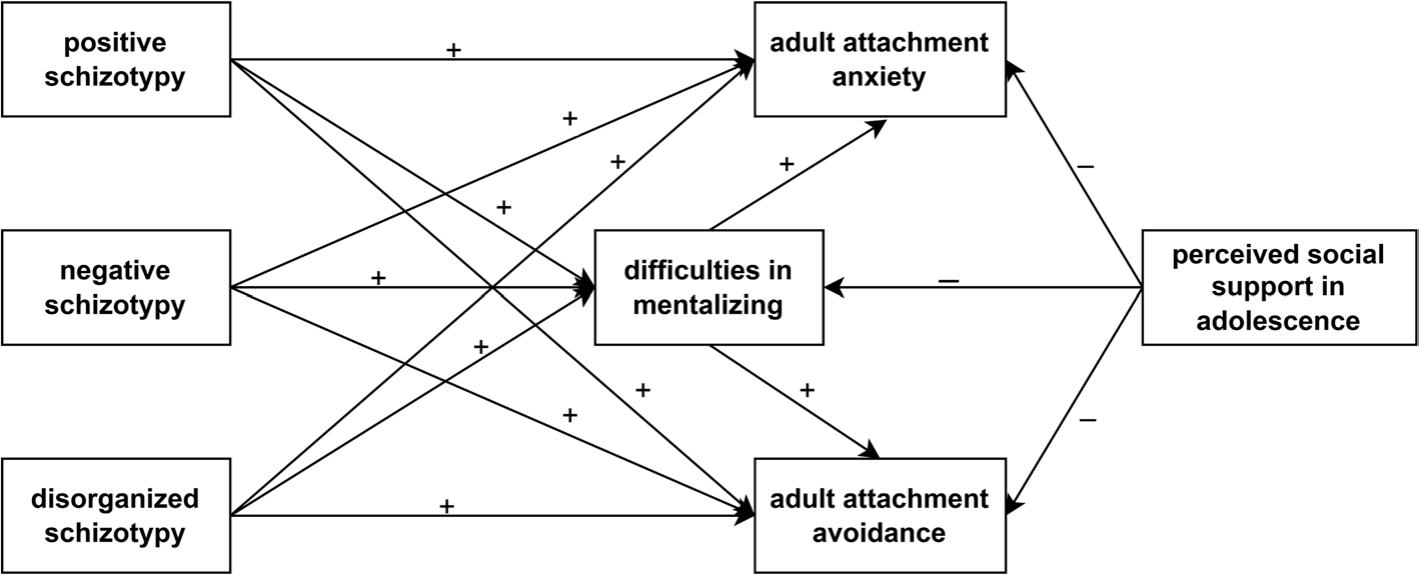
Model B to test the mediating effects of difficulties in mentalizing. Note: Arrows indicate hypothesized direct and indirect effects. Input variables include positive, negative, and disorganized schizotypal traits and perceived social support in adolescence. Outcome variables include adult attachment anxiety and avoidance. Control variables such as gender, age, and years of education are not shown in the figure to maintain readability. We hypothesized that positive, negative, and disorganized schizotypal traits contribute to higher levels of adult attachment anxiety and avoidance via difficulties in mentalizing. Conversely, perceived social support in adolescence was assumed to be negatively associated with difficulties in mentalizing as well as attachment anxiety and avoidance

In Model B (Fig. 3), predictor variables included positive, negative, and disorganized schizotypy subscale scores of the MSS-B, and MSPSS total score as a measure of perceived social support in adolescence. Similarly to Model A, the mediator was difficulties in mentalizing (MZQ total score), and outcome variables included adult attachment anxiety and avoidance (AAS scores). Gender, age, and years of education were controlled.

In both models, we used robust indirect effect size indicators to assess the mediating role of difficulties in mentalizing between predictor and outcome variables. A robust Maximum Likelihood Ratio (MLR) method was used to estimate model parameters due to the non-normal distribution of the data.

## Results

**Fig. 4:**
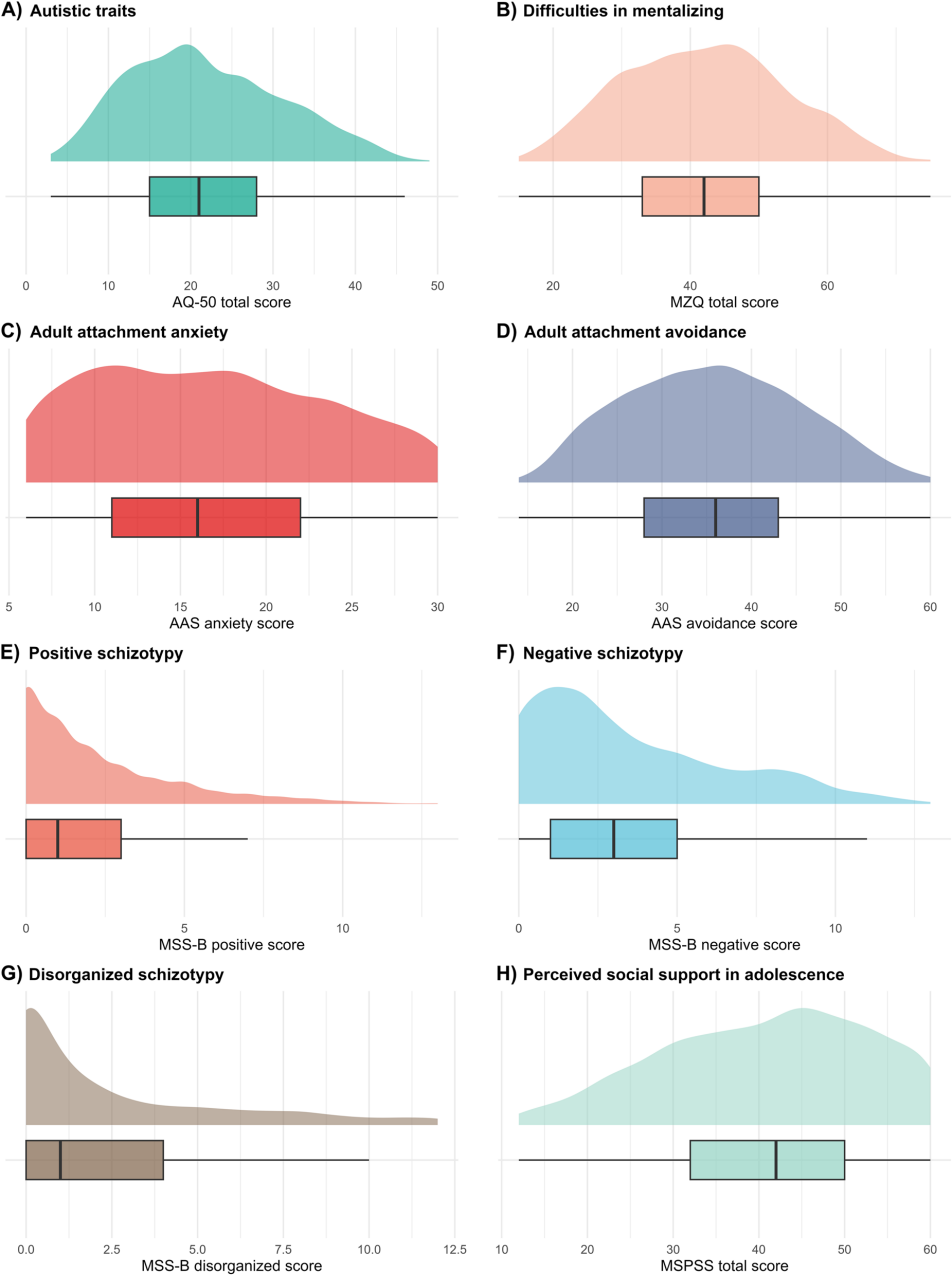
Distribution plots of questionnaire scores (*N* = 2203). Note: Boxes show the interquartile range, vertical lines indicate the median and the whiskers show the maximum and minimum values. Abbreviations: AQ-50 = Autism-Spectrum Quotient; MZQ = Mentalization Questionnaire; AAS = Adult Attachment Scale; MSS-B = Multidimensional Schizotypy Scale – Brief; MSPSS = Multidimensional Scale of Perceived Social Support

**Fig. 5:**
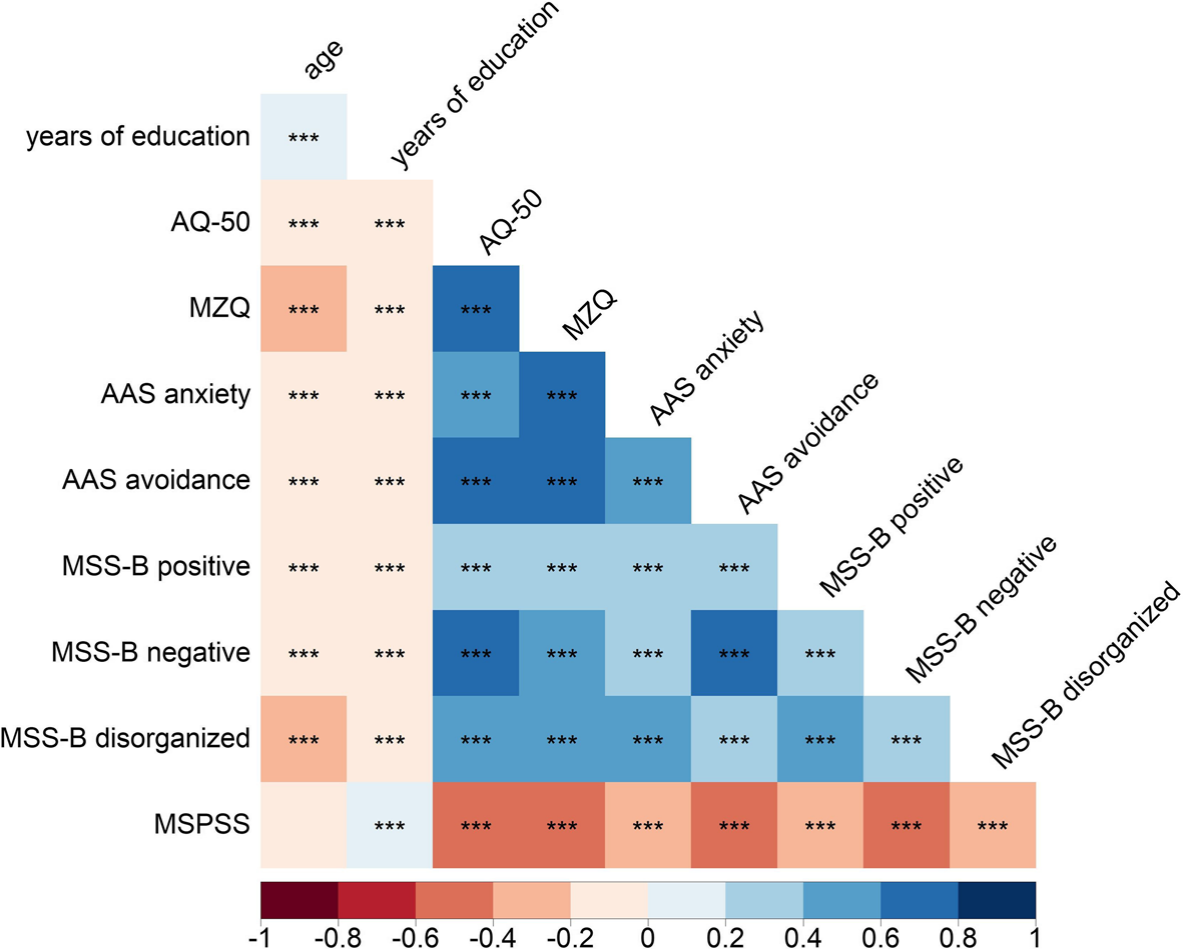
Correlation matrix of observed variables (*N* = 2203). Note: Numbers represent Spearman’s rank correlation coefficients (*r*_*S*_). Blue shades indicate positive, while red shades indicate negative correlations. Darker colors represent higher coefficients. Empty squares denote non-significant correlations; *** FDR-corrected *p* < 0.001. Importantly, moderate to strong positive correlations were observed between AQ-50 and AAS anxiety, AAS avoidance, MSS-B negative, and MSS-B disorganized, which suggested a significant overlap between autistic and schizotypal dimensions. High multicollinearity was observed between AQ-50 and MSS-B negative and disorganized subscales, which justified the separate testing of the two mediation models. MSPSS exhibited a weak to moderate negative correlation with AQ-50, MZQ, AAS anxiety, AAS avoidance, and MSS-B subscales, suggesting the protective nature of perceived social support in adolescence. Abbreviations: AQ-50 = Autism-Spectrum Quotient; MZQ = Mentalization Questionnaire; AAS = Adult Attachment Scale; MSS-B = Multidimensional Schizotypy Scale – Brief; MSPSS = Multidimensional Scale of Perceived Social Support

Table 2 shows the main descriptive statistics of all the questionnaire scores. The visualized distributions of the aggregated questionnaire scores can be seen in Fig. 4. A correlation matrix between variables is displayed in Fig. 5.

**Table 2:** Descriptive statistics of the questionnaire scores

	Minimum	Maximum	Mean	Median	Standard Deviation	Skewness
AQ-50	3	49	21,89	21	9,19	0,35
MZQ	15	75	42,01	42	11,64	0,12
AAS anxiety	6	30	16,7	16	6,69	0,22
AAS avoidance	14	60	35,75	36	9,64	0,13
MSS-B positive	0	13	2,12	1	2,44	1,47
MSS-B negative	0	13	3,5	3	3,04	0,86
MSS-B disorganized	0	12	2,55	1	3,25	1,31
MSPSS	12	60	40,81	42	11,94	-0,3

*Abbreviations*: *AQ-50* Autism-Spectrum Quotient, *MZQ* Mentalization Questionnaire, *AAS* Adult Attachment Scale, *MSS-B* Multidimensional Schizotypy Scale – Brief, *MSPSS* Multidimensional Scale of Perceived Social Support

**Fig. 6:**
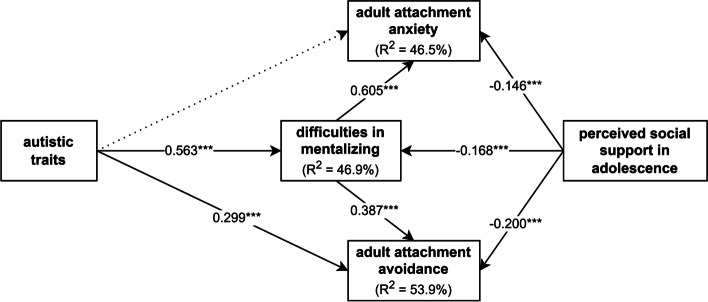
Results of mediation Model A. Note: Difficulties in mentalizing mediated the associations between autistic traits and perceived social support in adolescence as input variables and adult attachment anxiety and avoidance as outcome variables. A higher prevalence of autistic traits was linked to more severe difficulties in mentalizing, which were related to higher levels of adult attachment anxiety and avoidance. Conversely, higher levels of perceived social support in adolescence were associated with less severe difficulties in mentalizing, related to a more moderate level of adult attachment anxiety and avoidance. Standardized regression coefficients (β) are shown on the arrows. Continuous lines indicate significant, and dotted lines indicate non-significant regression effects. R^2^ = explained variance of the variable; *** *p* < 0.001

Figure 6 shows the results of Model A mediation analysis. A significant and strong relationship was observed between autistic traits and difficulties in mentalizing. In contrast, a significant weak negative association was found between the level of perceived social support in adolescence and difficulties in mentalizing. A strong significant association was found between difficulties in mentalizing and adult attachment anxiety, and a significant moderate positive relationship was found with attachment avoidance. Additionally, autistic traits showed a significant positive moderate direct relationship with adult attachment avoidance. Weak negative direct associations were found between perceived social support during adolescence and adult attachment anxiety and avoidance.

We found a moderate significant indirect effect of autistic traits and a small significant indirect effect of perceived social support in adolescence on adult attachment anxiety through difficulties in mentalizing. It is important to highlight that there was full mediation between autistic traits and attachment anxiety through difficulties in mentalizing. There were also significant weak indirect associations between adult attachment avoidance and autistic traits, as well as perceived social support in adolescence. Indirect effect size indicators are detailed in Table 3.

**Table 3:** Detailed total, direct, and indirect effect size indicators of mediation analyses

**MODEL A**				
**Predictor variables**	**Outcome variables**			
	**Attachment anxiety**		**Attachment avoidance**	
	B	β	B	β
**Autistic traits**				
Total effect	0.248***	0.341***	0.541***	0.517***
Direct effect	—	—	0.313***	0.299***
Indirect effect (via difficulties in mentalizing)	0.248***	0.341***	0.228***	0.218***
Indirect effect (in % of total effect)		100%		42.2%
**Perceived social support in adolescence**				
Total effect	-0.139***	-0.248***	-0.215***	-0.265***
Direct effect	-0.082***	-0.146***	-0.162***	-0.200***
Indirect effect (via difficulties in mentalizing)	-0.057***	-0.102***	-0.053***	-0.065***
Indirect effect (in % of total effect)		41.1%		24.5%
**MODEL B**				
**Predictor variables**	**Outcome variables**			
	**Attachment anxiety**		**Attachment avoidance**	
	B	β	B	β
**Positive schizotypy**				
Total effect	0.098**	0.036**	0.246**	0.062**
Direct effect	—	—	0.139*	0.035*
Indirect effect (via difficulties in mentalizing)	0.098**	0.036**	0.107**	0.027**
Indirect effect (in % of total effect)		100%		43.5%
**Negative schizotypy**				
Total effect	—	—	1.404***	0.441***
Direct effect	—	—	1.066***	0.335***
Indirect effect (via difficulties in mentalizing)	—	—	0.338***	0.106***
Indirect effect (in % of total effect)		—		24.0%
**Disorganized schizotypy**				
Total effect	0.613***	0.300***	0.354***	0.120***
Direct effect	0.161***	0.079***	-0.143*	-0.048*
Indirect effect (via difficulties in mentalizing)	0.452***	0.221***	0.497***	0.168***
Indirect effect (in % of total effect)		73.6%		—
**Perceived social support in adolescence**				
Total effect	-0.153***	-0.274***	-0.218***	-0.269***
Direct effect	-0.090***	-0.161***	-0.148***	-0.183***
Indirect effect (via difficulties in mentalizing)	-0.063***	-0.113***	-0.070***	-0.086***
Indirect effect (in % of total effect)		41.2%		32.0%

Blank cells did not prove significant

B = unstandardized regression coefficients; β = standardized regression coefficients

***p < 0.001, **p < 0.01, *p < 0.05

To summarize results yielded by Model A, difficulties in mentalizing mediated the effects of autistic traits and perceived social support during adolescence on adult attachment anxiety and avoidance. Specifically, more prevalent autistic traits were associated with more difficulties in mentalizing, which, in turn, corresponded to higher levels of adult attachment anxiety and avoidance. On the other hand, a greater amount of perceived social support during adolescence was related to fewer difficulties in mentalizing, resulting in lower levels of attachment anxiety and avoidance in adulthood.

**Fig. 7:**
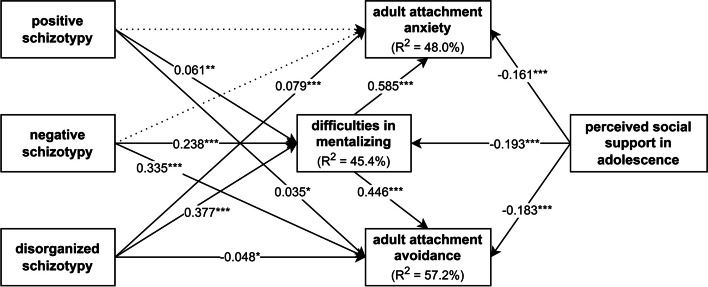
Results of mediation Model B. Note: More prevalent positive, negative, and disorganized schizotypal traits were associated with higher levels of adult attachment avoidance (and attachment anxiety for positive and disorganized schizotypy), partially through the mediating effects of difficulties in mentalizing. Conversely, higher levels of perceived social support in adolescence were associated with less severe difficulties in mentalizing, as well as lower attachment anxiety and avoidance. Standardized regression coefficients (β) are shown on the arrows. Continuous lines indicate significant, and dotted lines indicate non-significant regression effects. R^2^ = explained variance of the variable; *** *p* < 0.001; ** *p* < 0.01; * *p* < 0.05

Results from Model B mediation analysis are shown in Fig. 7. Positive schizotypal traits showed a significant weak direct association with difficulties in mentalizing as well as adult attachment avoidance. Negative schizotypy exhibited a significant weak positive relationship with difficulties in mentalizing, and a significant moderate positive direct relationship with attachment avoidance. However, the association between negative schizotypy and attachment anxiety did not prove to be significant. Disorganized schizotypal traits exerted significant weak direct effects on adult attachment anxiety, and significant moderate positive effects on difficulties in mentalizing. A somewhat unexpected finding was the significant negative direct relationship between adult attachment avoidance disorganized schizotypal traits. Nevertheless, considering the strength and direction of the total effect, this can be considered a statistical artifact. Additionally, we found significant weak negative direct associations between perceived social support during adolescence and difficulties in mentalizing, as well as adult attachment anxiety and avoidance.

Positive and disorganized schizotypy, as well as perceived social support during adolescence, showed a significant weak association with adult attachment anxiety, mediated by difficulties in mentalizing. Adult attachment avoidance showed weak indirect relationships with positive, negative, and disorganized schizotypal traits, as well as perceived social support in adolescence. Specific indirect effect size indicators are detailed in Table 3.

Overall, in Model B, difficulties in mentalizing fully mediated the effect of positive schizotypy on adult attachment anxiety. Furthermore, partial mediation was observed through mentalizing in the case of disorganized schizotypy and perceived social support in adolescence. Similarly, partial mediation was found through mentalizing between positive and negative schizotypy, perceived social support during adolescence as predictor variables, and adult attachment avoidance as the outcome variable. A full mediation was observed between disorganized schizotypy and attachment avoidance through difficulties in mentalizing. Specifically, higher levels of positive, negative, and disorganized schizotypy were associated with more severe difficulties in mentalizing, thereby leading to more prevalent adult attachment avoidance (and attachment anxiety for positive and disorganized schizotypal traits). In contrast, higher perceived social support was associated with less prevalent difficulties in mentalizing, resulting in lower adult attachment anxiety and avoidance.

## Discussion

The present study aimed to investigate the similarities between autism and schizophrenia spectrum by exploring difficulties in mentalizing and attachment, which may be key to uncovering their shared developmental roots. By taking a dimensional perspective, we measured autistic and schizotypal features as extended, disorder-liability traits of ASD and SCH. Our results provided evidence that autistic and schizotypal traits are associated with impairments in mentalizing and insecure adult attachment in comparable ways.

Previous research has consistently found deficits in mentalizing and a higher prevalence of insecure attachment styles in adulthood in both ASD and SCH [28, 57, 61]. Further, perceived social support during social development was suggested to be a key protective factor [51, 52]. Our results show that autistic and schizotypal traits might be risk factors for insecure adult attachment (higher anxiety and avoidance), through difficulties in mentalizing. More specifically, while autistic traits seem to mainly contribute to adult attachment anxiety through challenges in mentalizing, negative schizotypy relates to attachment avoidance, and disorganized schizotypal traits are linked to attachment anxiety. Conversely, perceived social support in adolescence was negatively linked to impairments in mentalizing and insecure adult attachment, serving as a protective factor during social-cognitive development.

Autistic traits were found to be linked to difficulties in mentalizing and insecure adult attachment, consistent with prior research revealing mentalizing impairments in autistic individuals [30, 83, 84]. Importantly, we found a stronger direct than indirect link between autistic traits and adult attachment avoidance. Conversely, the link between autistic traits and attachment anxiety was more indirect, with mentalizing fully mediating this relationship. This aligns partially with previous findings on healthy adults, which demonstrated the mediating effect of lower empathy between autistic traits and attachment avoidance [56]. However, this previous study only found a direct association between autistic traits and attachment anxiety. The divergence in findings may stem from methodological differences, as the previous study exclusively assessed one facet of mentalizing, empathy, using the unidimensional Empathy Quotient–Short [85], which focuses on inferring the emotional states of others [27]. In contrast, we employed the comprehensive Mentalization Questionnaire [69], covering four domains of mentalizing impairments. Additionally, the prior study employed a smaller sample size and different measures, specifically, the Broad Autism Phenotype Questionnaire [86] for autistic traits and the Experiences in Close Relationships–Revised [87] for attachment. Another study demonstrated a specific relationship between autistic traits and adult attachment avoidance in a normative sample [57], consistent with our findings. Here, difficulties in mentalizing fully explained the relationship between autistic traits and attachment anxiety, whereas attachment avoidance showed only partial mediation. This suggests that individuals with more prevalent autistic traits experience increased anxiety in interpersonal relationships due to difficulties in mentalizing.

The three dimensions of schizotypy (positive, negative, and disorganized) showed distinct associations with mentalizing and adult attachment. Positive schizotypy showed a weak association with adult attachment anxiety and avoidance through difficulties in mentalizing, consistent with the findings of a recent meta-analysis [88]. Positive schizotypy is also directly linked to adult attachment anxiety and avoidance, aligning with previous literature [61]. However, a large-scale network analysis [68] failed to identify positive traits as a central component in the relationship between schizotypy, mentalizing, and attachment. These results are unexpected, given that in cases of clinical SCH, paranoid delusions and hallucinations have been linked to deficits in mentalizing [34, 89, 90]. However, this inconsistency may be resolved by the “happy schizotypy” concept [91], which posits a subgroup characterized by positive schizotypal traits, high adaptation and creativity, and low scores on negative and disorganized dimensions [92]. Since our participants mostly lacked a psychiatric diagnosis of SCH, we can assume that positive schizotypal traits entail fewer mentalizing difficulties in their case.

Negative schizotypal traits were found to be related to adult attachment avoidance through difficulties in mentalizing, aligning with previous literature [88, 93, 94]. Furthermore, a study identified an association between negative schizotypy and avoidant attachment in samples of healthy young adults from the USA and Spain [95]. Additionally, a recent network analysis of autistic and schizotypal traits in the context of social functioning revealed a substantial overlap between autistic traits and negative schizotypy [40], both of which were found to be similarly linked to difficulties in mentalizing in our models. It is conceivable that individuals characterized by high levels of negative schizotypy exhibit difficulties in the motivational aspect of social engagement in the context of mentalizing and attachment, with decreased responsiveness to social stimuli. Disorganized schizotypy was associated with both adult attachment anxiety and avoidance, mediated by difficulties in mentalizing. Previous research suggested that disorganized traits are a central component of schizotypy [96], are associated with poorer cognitive and emotional control functions [97], and inversely relate to mentalizing [68]. Consequently, individuals with higher levels of disorganized schizotypy may experience difficulties in maintaining and tracking conversations with others, as well as integrating social cues from various sources (e.g., gestures, facial expressions, tone of voice, and verbal content). This may further complicate multi-actor interactions, leading to cumulative mentalizing errors without successfully coping with their emotional consequences due to impaired affective control functions. Repeated negative experiences in social interactions can result in attachment anxiety, and, particularly when associated with negative traits, avoidance of intimate relationships.

Perceived social support in adolescence showed a negative relationship with both difficulties in mentalizing and insecure adult attachment (anxiety and avoidance). This finding is consistent with previous literature that identified social support as a protective factor in the outcome of mental disorders [52]. Perceived social support enhances positive emotions, reduces negative ones, and contributes to overall life satisfaction, thereby contributing to subjective well-being [51]. Some authors suggest that social support directly impacts general mental health, while others consider it a specific resource that promotes coping with stress [73]. Within the mentalizing approach, it appears that perceived social support provides an environment that facilitates epistemic trust, which is our pre-programmed ability to perceive others as reliable sources of information, thereby expanding our knowledge of the world [28]. Epistemic trust contributes to the development of flexible mentalizing, and accordingly, perceived social support in adolescence can be considered a protective factor against adult difficulties in mentalizing and insecure attachment.

Summarizing the above results, both autistic and schizotypal traits appear to play a crucial role in the development of mentalizing difficulties and insecure adult attachment. The results might suggest a developmental trajectory in which autistic and schizotypal traits reflect atypical neurodevelopment [4–7], a liability for ASD and SCH, respectively. These features might influence social-cognitive development throughout life, resulting in ineffective mentalizing, further consolidating attachment anxiety, and avoidance [28]. However, it is an area for future longitudinal research to confirm these possible trajectories, as our study utilized cross-sectional data. Our findings seem to align with previous research on the neural underpinnings of ASD and SCH, highlighting their similarities. Mentalizing difficulties and anxious-avoidant attachment in ASD may be due to atypical amygdala functioning and connectivity [83, 98], as the neural circuits connecting the amygdala and the prefrontal cortex play a key role in balanced mentalizing [29]. Similarly, individuals with SCH exhibit altered threat processing [99] and decreased emotion recognition, also linked to atypical amygdala functioning [100, 101]. We could hypothesize that individuals with more prevalent autistic and schizotypal traits may experience inadequate amygdala activation, less controlled mentalizing, increased anxiety, and mentalizing errors in interpersonal situations. In the long term, this may result in the development of more insecure attachment styles, specifically, anxious attachment, in adulthood. However, as our study did not include neuroimaging to measure amygdala activation, we propose these notions as areas for future research that should combine self-report and neural measures. Additionally, it may be beneficial to conduct separate analyses for the clinical population after further data collection, as the proposed relationships could exhibit different patterns (e.g. quadratic) across the autistic and schizotypal spectrum.

Our findings are an important contribution to the literature which considers psychopathological phenomena from a dimensional perspective. This approach has shown greater reliability and validity compared to categorical, diagnosis-based measures [26]. However, a limitation lies in the cross-sectional nature of the study, which hinders the possibility to draw causal inferences and calls for longitudinal and experimental designs to validate our results. Additionally, the use of online self-report questionnaires may have biased the final composition of our sample, as individuals at the more severe end of psychopathology dimensions may have failed to fill out the questionnaires completely, resulting in dropout due to incomplete scores. Furthermore, self-reports might be biased by the participants’ subjective views of themselves. Moreover, the use of online assessment methods, and socioeconomic makeup of our sample further limit the generalizability of our results, as individuals with greater support needs (potentially lacking internet access) could not be included in our study. Considering the diagnostic composition of our sample, there is an overrepresentation of individuals with ASD compared to the general population. Additionally, our sample is likely skewed due to the recruitment of patients primarily through outpatient units, where individuals are more likely to be actively seeking help. The gender demographics of our sample also do not reflect those of the autistic population, with our sample predominantly consisting of females, whereas autistic populations typically have a male-to-female ratio of two to three times higher [31]. Additionally, elevated AQ-50 and MSS-B scores indicate autistic and schizotypal traits rather than being specific to ASD or SCH, cautioning against concluding that our findings are specific to these conditions [7, 63]. While we acknowledge that some findings may overlap with general psychopathological frameworks [102], our goal was to contribute to a more detailed understanding of how these features manifest uniquely or similarly within these conditions. Furthermore, our models did not differentiate between difficulties in dimensions of mentalizing (e.g., cognitive or affective), suggesting another area for future research.

Our findings have important practical implications for the support of individuals with ASD and SCH spectrum disorders. For example, the characteristics of attachment highly influence the patient-therapist collaboration in psychotherapy [103], as well as adherence to pharmacotherapy [104, 105]. Considering the specific mentalizing and attachment characteristics of individuals enables more personalized and effective healthcare, as these interventions necessarily occur through social interactions. Additionally, mentalizing can serve as a target of novel therapeutic interventions which aim to ameliorate the social functioning of individuals, as there is evidence for the efficacy of mentalization-based interventions in both ASD [44, 45] and SCH [42, 43].

## Conclusions

In conclusion, our findings on the effect of mentalizing and social support on adult attachment emphasize the essential role of supportive therapeutic relationships and community care in autism and schizophrenia spectrum conditions. Overall, this study has contributed to a deeper understanding of autistic and schizotypal dimensions as well as their overlaps and could take us one step further towards understanding the brain correlates and developmental roots of ASD and SCH.

## Data Availability

The datasets used and analyzed during the current study are available from the corresponding author on reasonable request.

## Abbreviations

AAS: Adult Attachment Scale
AQ-50: Autism-Spectrum Quotient
ASD: Autism spectrum disorder
FDR: False discovery rate
MLR: Maximum likelihood ratio
MSPSS: Multidimensional Scale of Perceived Social Support
MSS-B: Multidimensional Schizotypy Scale – Brief
MZQ: Mentalization Questionnaire
SCH: Schizophrenia spectrum disorder

## Data availability

The data that support the findings of this study are available from the corresponding author upon request.
